# Progression of coronary microvascular dysfunction to heart failure with preserved ejection fraction: a case report

**DOI:** 10.1186/s13256-019-2074-z

**Published:** 2019-05-06

**Authors:** Sandy Joung, Janet Wei, Michael D. Nelson, Haider Aldiwani, Chrisandra Shufelt, Balaji Tamarappoo, Daniel Berman, Louise E. J. Thomson, C. Noel Bairey Merz

**Affiliations:** 1 Barbra Streisand Women’s Heart Center, Cedars-Sinai Heart Institute, 127 S. San Vicente Blvd, Suite A3600, Los Angeles, CA 90048 USA; 2 Applied Physiology and Advanced Imaging Laboratory, Arlington, TX 76019 USA; 3 Biomedical Imaging Research Institute, Cedars-Sinai Medical Center, Los Angeles, CA 90048 USA

**Keywords:** Coronary microvascular dysfunction, Heart failure with preserved ejection fraction, Non-obstructive coronary artery disease, Cardiac magnetic resonance imaging

## Abstract

**Background:**

In women with evidence of ischemia and no obstructive coronary artery disease the underlying mechanism is most often attributed to coronary microvascular dysfunction. Higher rates of adverse cardiovascular events, specifically heart failure with preserved ejection fraction, are present in women with coronary microvascular dysfunction, leading to the hypothesis that coronary microvascular dysfunction may contribute to the progression of heart failure with preserved ejection fraction.

**Case summary:**

A 55-year-old, Caucasian woman with a past medical history of chest pain and shortness of breath was referred to our tertiary care center and diagnosed as having coronary microvascular dysfunction by invasive coronary reactivity testing. After 10 years of follow-up care for coronary microvascular dysfunction, she presented to an emergency room in acute heart failure and was diagnosed as having heart failure with preserved ejection fraction.

**Discussion:**

The current case report provides a specific example in support of existing studies that demonstrate that coronary microvascular dysfunction may be a precursor of heart failure with preserved ejection fraction. Further research is needed to establish causality and management.

**Trial registration:**

Clinical Trial Registration: ClinicalTrials.gov Identifier: NCT02582021.

## Introduction

Cardiovascular disease remains the number one cause of death in the USA [1]. Coronary microvascular dysfunction (CMD) is common in women with evidence of ischemia and no obstructive coronary artery disease (INOCA) [2]. Previous studies have shown that these women have a higher risk of fatal and non-fatal cardiovascular events in comparison to asymptomatic healthy women and are more likely to be readmitted for angina or for acute coronary syndrome within 180 days of having normal coronary angiography [3]. These women also experience unexpectedly higher rates of heart failure hospitalizations [3]. Remarkably, a previous report from the Women’s Ischemia Syndrome Evaluation (WISE) study revealed that in women with signs and symptoms of INOCA hospitalized for heart failure, 90% had preserved left ventricular ejection fraction (LVEF). Although the prevalence of CMD in heart failure with preserved ejection fraction (HFpEF) is currently unknown, exercise studies have indicated that vascular stiffness, impaired exercise vasodilation, and impaired diastolic reserve may be related to endothelial and microvascular dysfunction [4]. This has led to the hypothesis that CMD may contribute to the development of HFpEF [5]. The following case highlights some of the first empirical evidence supporting this hypothesis.

This case demonstrates that progression to HFpEF may occur parallel with common pathophysiological mechanisms or risk factors of CMD. To date, there has been indirect and limited clinical evidence of CMD as a novel mechanism underlying the pathogenesis of HFpEF. This case underscores the importance of investigating the link between these two conditions. Understanding the correlates associating CMD in HFpEF could provide additional insight into the mechanisms of HFpEF, improve health outcomes for patients with CMD, and help in the design and development of future preventative pharmacotherapies.

## Case presentation

### Initial presentation

A 55-year-old, Caucasian woman was referred to our tertiary women’s heart center for persistent chest pain, palpitations, and dyspnea. Her medical history included hypertension, dyslipidemia, chronic anxiety, and bilateral non-obstructive carotid atherosclerosis. She had no prior history of diabetes mellitus, tobacco smoking, alcohol or substance abuse, or adverse pregnancy outcomes. Her family history was significant for premature coronary artery disease. Her father had a history of hypertension and had a myocardial infarction (MI) and coronary artery bypass grafting at the age of 39. Her brother had a history of coronary artery disease and also had a MI at the age of 40. Her occupational history indicated that she had been working in the field of psychology and was still an employee in the same job at the time of the hospital visit and follow-up care.

Table 1 summarizes the general symptoms and characteristic signs of our patient for the onset of CMD and her progression to HFpEF. She had undergone an exercise treadmill test which revealed ischemic ECG changes and dyspnea. Her initial echocardiogram demonstrated a LVEF of 67%, mild diastolic dysfunction, mild left ventricular (LV) hypertrophy, no significant valvular heart disease, and no pulmonary hypertension. Subsequent invasive left heart catheterization was performed and it showed normal epicardial coronary arteries without angiographic evidence of atherosclerotic plaque. She continued to have exertional symptoms and angina-like chest pain and was subsequently referred to our center for further evaluation of suspected INOCA. During her evaluation and treatment she continued to experience stable angina and exertional dyspnea despite initial management with atorvastatin 20 mg daily, lisinopril 20 mg daily, aspirin 81 mg daily, and sublingual nitroglycerin as needed. She had a poor clinical response to sublingual nitroglycerin. Due to her persistent symptoms and abnormal stress testing, she was referred for coronary reactivity testing (CRT) to establish the diagnosis of CMD.

**Table 1:** Timeline of coronary microvascular disease onset, progression to heart failure with preserved ejection fraction, and therapy

Time	Visit type	Symptoms	Medications	Vital signs	Laboratory assessments	Diagnostic testing
December 2006 (baseline)	Initial evaluation for symptoms of ischemic heart disease	5-month history of dyspnea both at rest and at exertion, squeezing chest pain on a daily basis, intermittent palpitations	Lipitor (atorvastatin; 20 mg, daily), lisinopril (20 mg, daily), aspirin (81 mg, daily) and sublingual nitroglycerin (0.4 mg, as needed)	Blood pressure, 120/75 mmHg; pulse, 60 bpm	Sodium, 143 (reference range, 135–145 mmol/L); potassium, 3.8 (reference range, 3.5–5.0 mmol/L), creatinine, 0.5 (0.6–1.1 mg/dL)	Diagnostic coronary reactivity testing demonstrating coronary endothelial dysfunction. Previous right left heart catheterization showed normal coronary arteries and was negative for any shunt. CMRI showed normal left ventricular structure and function. MPRI borderline normal 1.8
November 2016 (10-year follow-up)	Emergency Department visit for heart failure and initial diagnosis for heart failure with preserved ejection fraction	Increased orthopnea, dyspnea, mild diffuse headache, lower extremity edema, and elevated blood pressure	Eplerenone (25 mg, daily), lisinopril (40 mg, daily), aspirin (81 mg, daily), pravastatin (40 mg, daily), spironolactone (100 mg, daily), nitroglycerin (0.4 mg, as needed)	Blood pressure, 152/77 mmHg; pulse, 70 bpm; respiratory rate, 18; temperature, 36.9 °C (98.4 °F)	BNP, 406 (reference range, < 100 pg/mL); troponin, < 0.01 (reference range, < 0.04 ng/mL); hemoglobin, 9.9 (reference range, 10.6–13.5 g/dL); sodium, 139 (reference range, 135–145 mmol/L); potassium, 4.3 (reference range, 3.5–5.0 mmol/L); creatinine, 0.5 (0.6–1.1 mg/dL)	Follow-up CMRI revealed worsening ischemia with MPRI 1.1 and increased wall thickness, with evidence of myocardial steatosis

*bpm* beats per minute, *BNP* brain natriuretic peptide, *CMRI* cardiac magnetic resonance imaging, *MPRI* myocardial perfusion reserve index

### Diagnosis of CMD

Our patient underwent invasive CRT, as previously published [6]. Testing demonstrated normal coronary flow reserve (CFR) in response to intra-coronary adenosine (CFR 3.1; normal ≥ 2.5), abnormal macrovascular endothelial function to intra-coronary acetylcholine (− 6% change in coronary diameter, constriction; normal, dilation), abnormal microvascular endothelial function (coronary blood flow change 48%; normal ≥ 50%), and abnormal non-endothelial function to intra-coronary nitroglycerin (coronary diameter change + 0%; normal dilation) (Table 2). She also underwent cardiac magnetic resonance imaging (CMRI) with perfusion imaging at rest and with adenosine stress (140 μg/kg per minute) which showed circumferential subendocardial perfusion defect at stress, normal LV end-diastolic volume indexed to body surface area (EDVi) of 56.4 mL/m^2^, LV mass index 42.3 grams/m^2^, and no LV hypertrophy (septum 7.2 mm and lateral wall 6.0 mm). The myocardial perfusion reserve index (MPRI) was 1.8 which was considered borderline abnormal [7] (Table 3). There was no evidence of myocardial scar.

**Table 2:** Results of coronary reactivity testing

Coronary microvascular dysfunction pathways	Microvascular dysfunction	Macrovascular dysfunction
Non-endothelial dependent	^a^Coronary flow reserve in response to adenosine 2.8 (normal ≥ 2.5)	^b^Change in coronary diameter in response to nitroglycerin 0% (normal ≥ 20%)
Endothelial dependent	^c^Change in coronary blood flow in response to acetylcholine 48% (normal ≥ 50%)	^d^Change in coronary diameter in response to acetylcholine − 6% (normal ≥ 5%)

^a^ Non-endothelial-dependent microvascular dysfunction with coronary flow reserve 2.8 (adenosine). ^b^ Non-endothelial-dependent macrovascular dysfunction with 0% (nitroglycerin) coronary diameter change. ^c^ Endothelial-dependent microvascular dysfunction with coronary blood flow change 48%. ^d^ Endothelial-dependent macrovascular dysfunction with coronary diameter change − 6% in response to acetylcholine

**Table 3:** Changes in left ventricular morphology

Cardiac magnetic resonance imaging parameters	Baseline	10 years later
LV EDVi (mL/m^2^)	56.4	69
LV ESVi (mL/m^2^)	20.8	23.6
LVMi (g/m^2^)	42.3	48.5
LV mass-to-volume ratio (g/mL)	0.75	0.70
Wall thickness		
Septum (cm)	7.15	9.34
Lateral wall (cm)	6.06	7.06
Scar (g)	0	0
LVEF (%)	67	64
LGE	Yes	Yes
MRPI	1.8	1.1

*EDVi* end-diastolic volume indexed to body surface area, *ESVi* end-systolic volume indexed to body surface area, *LGE* late gadolinium enhancement, *LV* left ventricular, *LVEF* left ventricular ejection fraction, *LVMi* left ventricular mass indexed to body surface area, *MPRI* myocardial perfusion reserve index

The diagnosis of CMD was established by the coronary endothelial dysfunction observed with invasive CRT, and carvedilol and eplerenone 25 mg daily were added to her regimen. She was followed regularly in clinic with good control of her blood pressure and serum lipid levels. She reported improvement of her angina and dyspnea along with reduction in the duration and frequency of these episodes.

### Diagnosis of heart failure

Ten years after her initial diagnosis of CMD, our patient was hospitalized due to symptoms of dyspnea. She was found to have elevated brain natriuretic peptide (BNP) levels of 406 pg/mL and normal LVEF. She had a computed tomography (CT) angiogram of her chest to evaluate for pulmonary embolism, which was negative but revealed bilateral pulmonary edema. She was treated with intravenously administered furosemide for pulmonary edema and diagnosed as having HFpEF. Subsequently, she was discharged with instructions to increase her eplerenone.

She continued to experience worsening dyspnea on exertion, orthopnea, and paroxysmal nocturnal dyspnea. A repeat echocardiogram demonstrated normal LV systolic function with an LVEF of 64%, and diastolic dysfunction as evidenced by decreased lateral E′ velocity (4.2 cm/s, indicating impaired myocardial relaxation) and elevated E/E′ ratio 12.9 (suggestive of increased LV filling pressure). She underwent coronary CT angiography which showed absence of coronary atherosclerotic plaque and a coronary calcium score of 0. She was diagnosed as having HFpEF based on clinical symptoms, preserved ejection fraction of 64%, elevated BNP, and evidence of diastolic dysfunction.

**Fig. 1:**
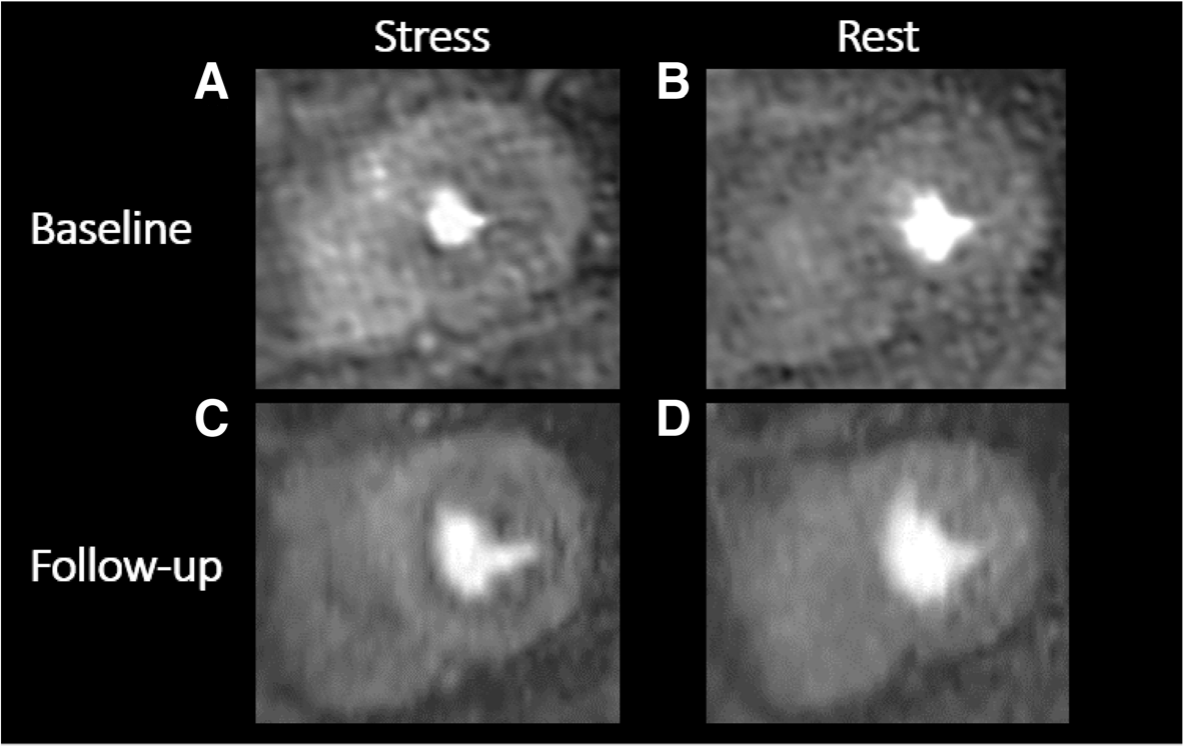
Baseline (**a, b**) and 10-year follow-up (**c, d**) adenosine stress first-pass perfusion cardiac magnetic resonance imaging showing evidence of circumferential subendocardial hypoperfusion at stress, consistent with coronary microvascular dysfunction-related ischemia. Myocardial perfusion reserve index decreased from 1.8 to 1.1 over 10-year period, indicating worsened ischemia

As part of the WISE – Coronary Vascular Dysfunction (WISE-CVD) Continuation Study (NCT00832702), she underwent a repeat rest-stress CMRI to assess myocardial structure, function, perfusion, and scar, and ^13^C magnetic resonance (CMR) spectroscopy. Compared to her prior CMRI 7 years ago, she had an increase in LV wall thickness in both the septum and lateral wall (Table 3). On CMR spectroscopy, the myocardial triglyceride content was elevated (0.83%) compared to normal control women (mean 0.43%), suggesting myocardial steatosis which is consistent with an ischemia-induced metabolic shift and HFpEF phenotype [8]. Adenosine stress first pass-perfusion CMRI again showed circumferential subendocardial hypoperfusion (Fig. 1) and her MPRI worsened from 1.8 to 1.1, consistent with severe CMD [7]. There was no evidence of scar on late gadolinium enhancement imaging.

## Discussion

This case documents, in a 55-year-old woman, the progression of CMD diagnosed with invasive CRT to HFpEF over a span of 10 years despite well-controlled hypertension and dyslipidemia. Although we and others have previously hypothesized that CMD may contribute to progression to HFpEF [9, 10], we are not aware of case examples of CMD progressing to HFpEF reported in the literature. While existing cross-sectional registry studies cannot determine whether primary CMD leads to ventricular remodeling, diastolic dysfunction, and HFpEF or whether these findings observed in HFpEF lead to secondary CMD [11], the current case example provides evidence that CMD may contribute to the development of HFpEF.

HFpEF has often been referred to as “diastolic heart failure” and is characterized by impaired LV relaxation and elevated LV filling pressures [12]. The diagnosis of HFpEF is challenging and currently accounts for approximately half of all cases of heart failure [12], with morbidity and mortality rates similar to those with heart failure with reduced ejection fraction [13, 14]. Previous research has observed an increased incidence of CMD as evidenced by abnormal CFR and index of microvascular resistance (IMR) after adenosine administration in patients with HFpEF compared with normal reference control individuals [15, 16].

At present, there are insufficient prospective studies to confirm that CMD contributes to progression to HFpEF, nor putative mechanistic pathways. Previous studies have identified common inflammatory markers as contributing to the progression to heart failure [17]. Systemic inflammation may lead to reduced nitric oxide bioavailability, expression of growth factor-β, activation of cardiac fibroblasts, and an increase in collagen type 1 formation [18]. In addition, this process may lead to interstitial fibrosis contributing to high diastolic LV stiffness and eventual progression to HFpEF [18]. There are currently no evidence-based treatments for CMD or HFpEF. Understanding the links and disease pathophysiology between CMD and HFpEF may lead to the development of preventive and treatment strategies [19].

## Conclusions

This case report of a patient with HFpEF and antecedent CMD is in line with our hypothesis that CMD may contribute to the development of HFpEF [5, 8]. In an individual patient, whether these are causally related, whether they are simply related to similar underlying risk factors, or whether they represent unrelated presences of two common disorders is the subject of current research. Further evaluation of the association between CMD and HFpEF will be necessary to prove our hypothesis.

## Abbreviations

BNP: Brain natriuretic peptide
CFR: Coronary flow reserve
CMD: Coronary microvascular dysfunction
CMR: ^13^C magnetic resonance
CMRI: Cardiac magnetic resonance imaging
CRT: Coronary reactivity testing
CT: Computed tomography
HFpEF: Heart failure with preserved ejection fraction
IMR: Index of microvascular resistance
INOCA: Ischemia and no obstructive coronary artery disease
LV: Left ventricular
LVEF: Left ventricular ejection fraction
MI: Myocardial infarction
MPRI: Myocardial perfusion reserve index
WISE: Women’s Ischemia Syndrome Evaluation
WISE-CVD: Women’s Ischemia Syndrome Evaluation – Coronary Vascular Dysfunction
